# Modern Medicine

**Published:** 1901-02

**Authors:** 


### Book Reviews.

---

MODERN MEDICINE. By JULIUS L. SALINGER, M. D., Demonstrator of Clinical Medicine, Jefferson Medical College; Hematologist to the Jefferson Medical College Hospital; Pathologist to the Lying-in Charity Hospital, Philadelphia; Assistant Pathologist to the Philadelphia Hospital. And Frederick J. Kalteyer M. D., Assistant Demonstrator of Clinical Medicine, Jefferson Medical College Hospital; Hematologist to the Jefferson Medical College Hospital. Octavo, pp. 801. Illustrated. Philadelphia and London: W. B. Saunders & Co. 1900. [Price, cloth, $4.00 net.]

It was a great task which Drs. Salinger and Kalteyer set out to perform when they undertook to produce a book on medicine, for at first glance, one would imagine that the field was just about full as it could conveniently be without crowding, yet they have done their work, and done it well. The result is, this volume, which is very properly called Modern Medicine, and it is no exaggeration to say that it is one of the most complete works of its kind which has yet appeared.

There are reasons for this statement, written with such works within easy reach as the gloomy yet exhaustive Osler, the interesting Tyson, the instructive Anders, and the incomparable Pepper system.

Modern Medicine is complete because it covers all the specialties in clinical medicine which are generally crowded out of the more pretentious works, and at the same time, does not slight any of the subjects which belong in such a work. This will be better understood when it is explained that at the outset the specialties are considered, and in such manner as to be wholly instructive as well as entertaining. Physical diagnosis, bacteriological examinations, gastric contents, and the examination of urine, blood, sputum and feces, are all well considered. The wonder is that some of the medico-literary bright lights have not thought of this combination before. It would certainly have helped along the practice of medicine, and made physicians more self-reliant, just as this book will serve to do.

As one gets along into the pages of Modern Medicine, the charm and the simplicity and the convenience of its arrangement becomes more and more apparent. Under clinical bacteriology, one finds all one cares to know in a general way concerning the pathogenic bacteria, which is very delightful for it removes the necessity of bothering one's head about anything but etiology, pathology, symptoms, diagnosis and treatment when the diseases are reached. That's one of the new things about Modern Medicine, too, when you come to think of it. And when you do finally get into the disease proper, you get another shock, if you've got any old fogyish ideas in your head. You find everything in a condensed form, which is readable, understandable and interesting. Really, it is almost like a story in some places, it is all so simple and yet so complete, for in spite of condensation, there is nothing of importance neglected or lacking. Instead of the page after page and page and page again and again of typhoid and tuberculosis, descriptive, explanatory, statistical, argumentative and pathologic beyond all hope of redemption, which one must wade through in some of the standard works, in Modern Medicine we have 12 pages of typhoid and 21 pages of tuberculosis, and the latter covers the disease in all its phases and in all the organs. Yet, in those pages devoted to the subject the authors have told everything of moment concerning the diseases from the beginning to the end. They do not take up space telling what they have done; they confine themselves to telling all about what the disease does and how properly to cure it.

Beginning with symptomatology and semeiology, they go on to physical diagnosis, clinical bacteriology and laboratory methods, and then Part I. looms into view with the infectious diseases. After this comes the diseases of the circulatory and respiratory systems; the digestive tract and kidneys; constitutional diseases; diseases of the blood and ductless glands, nervous system and muscles; intoxications and sun stroke, and diseases due to animal parasites, which with a very cleverly arranged index completes the book.

A few words are due the illustrations. There are not many, but those which are shown are in keeping with the literary portion of the book—they are excellent, novel in some respects and modern in every way. In no other work on general medicine will there be found such exquisite plates of the malarial parasite in all forms and conditions. These plates were colored and in themselves stamp the work as modern. Quite an interesting map of the world is shown, which by means of shading of various degrees shows where malaria is most prevalent. One of the darker spots, on North America is that part of the country immediately surrounding Lake Erie and Ontario, and the dark spot means malaria; but since these maps are credited to Mannaberg, Drs. Salinger and Kalteyer cannot be held responsible for this slight error of location which makes Buffalo a malaria center.

Summed all up, Modern Medicine is an ideal book, prepared by modern men with modern ideas who have that happy and also too rare faculty,of saying what they have to say about a disease and stopping when they've said it. This is the first edition. Others will follow as a necessity,for it is a work which in years to come will stand in single-volume state and dignity, beside the more pretentious and heavier volumes and systems of more expansive authors and editors.

